# *Tachinobia repanda* (Hymenoptera: Eulophidae) From Egg Sacs of a Colonial Spider, *Cyrtophora moluccensis* (Araneae: Araneidae) in Papua New Guinea

**DOI:** 10.1093/jisesa/ieaa104

**Published:** 2020-09-28

**Authors:** Zoya A Yefremova, Yael Lubin

**Affiliations:** 1 Department of Zoology, Steinhardt Museum of Natural History, Tel Aviv University, Tel Aviv, Israel; 2 Mitrani Department of Desert Ecology, Blaustein Institutes for Desert Research, Ben-Gurion University of the Negev, Sede Boqer Campus, Midreshet Ben-Gurion, Israel

**Keywords:** Tetrastichinae wasp, gregarious endoparasitoid, hyperparasitoid, sarcophagid fly

## Abstract

We report the discovery of the wasp *Tachinobia repanda* Bouček collected from egg sacs of the colonial spider *Cyrtophora moluccensis* (Doleschall) in Morobe Province, Papua New Guinea (PNG) by Lubin, Y.D. in 1980. This is the first record of *T. repanda* from egg sacs of a colonial spider. The likely host of this eulophid wasp was the larvae of a sarcophagid fly that parasitizes the egg sacs of these spiders. The 67 *T. repanda* collected were all females and varied little in body size. We suggest that this species is a gregarious hyperparasitoid.

Currently, six genera of Tetrastichinae wasps (Hymenoptera: Eulophidae: Tetrastichinae) are known from spider egg sacs: *Aprostocetus*, *Arachnoobius*, *Aranobroter, Baryscapus*, *Tachinobia*, and *Tetrastichus* (LaSalle 1990). *Aprostocetus* was mentioned as a predator on the eggs of *Araneus omnicolor* (Araneae: Araneidae) in Brazil (Sobczak et al. 2015), and *Tachinobia repanda* Bouček was reared from egg sacs of an unknown spider species in Cuba (LaSalle 1994).

The genus *Tachinobia* Bouček is a group of small gregarious parasitic wasps of Lepidoptera and Diptera (Bouček 1977, LaSalle 1994, Marchiori and Silva 2005, Marchiori 2006). There are currently three accepted species in the world: two from Africa, *Tachinobia diopsisephila* (Risbec) and *Tachinobia zairensis* Doğanlar and one from the Oriental region, *T. repanda* Bouček (Noyes 2020). There are few studies of the ecology of this genus (Bouček 1977, 1988; Marchiori and Silva 2005; Marchiori 2006).

*Tachinobia repanda* was described by Bouček (1977) from Papua New Guinea (PNG). The known distribution of this parasitoid wasp is Southeast Asia: PNG, Indonesia, Malaysia, India (Bouček 1977), Solomon Island, and Thailand (Bouček 1988). It was also found in the southern United States and Cuba, where it is likely invasive (Bouček 1977).

*Tachinobia repanda* is described as a gregarious pupal endoparasitoid of the flies, *Bessa remota* (Aldrich), *Blepharipa* sp., *Cadurcia* sp., and *Carcelia lagoae* (Townsend) (Diptera: Tachinidae), that attack caterpillars of *Papilio laglaizei* Depuiset (Lepidoptera: Papilionidae), and of *Sarcophaga banksi* Senior-White (Diptera: Sacophagidae) attacking larva of *Mahasena corbettii* Tams (Lepidoptera: Psychidae; Bouček 1977). If tachinid and sarcophagid larvae are endoparasitoids of butterfly and moth caterpillars, then *T. repanda* can be defined as a hyperparasitoid (Godfray 1994).

In this study, we provide additional morphological characters of *T. repanda* and new biological data about the associations between the spider *Cyrtophora moluccensis* (Doleschall), the parasitoid fly *Sarcophaga* (*Baranovisca*) *cyrtophorae* (Cantrell) (Diptera: Sarcophagidae) and the eulophid hyperparasitoid *T. repanda*.

## Material and Methods

***Tachinobia repanda*** was identified by comparing morphological characters of specimens with the original description by Bouček (1977) and using the keys to the genera of Tetrastichinae (LaSalle 1994) and to the genera of Eulophidae (Gibson et al. 1997).

Morphological terminology and abbreviations follow Gibson (1997). Abbreviations are the following: POL, postocellar length; OOL, ocular-ocellar length; SMV, MV, PMV, STV—submarginal, marginal, postmarginal, and stigmal veins. Absolute measurements in millimeters (mm) were used for the body and forewing length. For all other dimensions, relative measurements were used. Observations and measurements were taken using a Leica M 125. The photographs were taken in the Entomology Department, Museum of Comparative Zoology, Harvard University, Cambridge, USA.

**Parasitism rates of *C. moluccensis* egg sacs** are derived from 1980 field notes of YL from PNG. These data were collected as part of a study of the ecology of this colonial spider, conducted at the Wau Ecology Institute, Wau, PNG (−7°20′16.01″S, 146°42′59.36″E; Lubin 1974, 1980a,b).

## Results and Discussion

***Tachinobia repanda*** Bouček, 1977

*Tachinobia repanda* Bouček, 1977: 27.

Holotype, female, PNG, Central District, Konedobu, ex *Blepharipa* in *Papilio laglaizei,* 4.vii.1973 T. L. Fenner (BMNH, not examined).

### Diagnosis

**Figs. 1–4.**
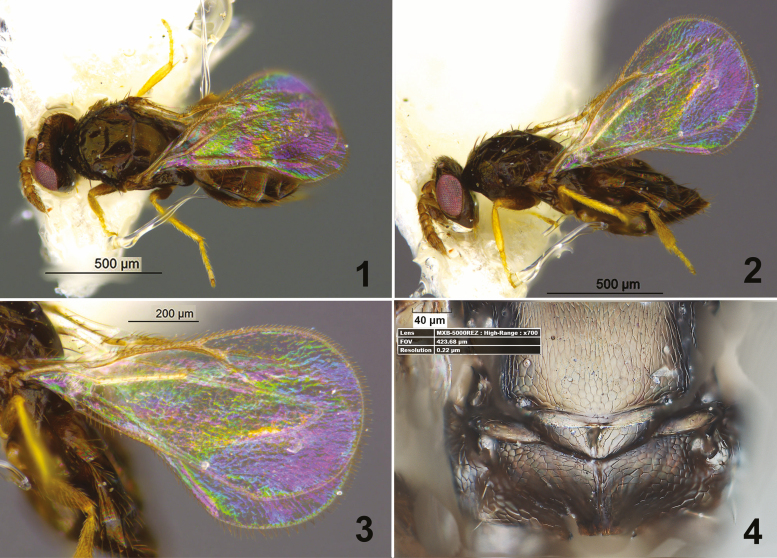
*Tachinobia repanda* Bouček. (1) Female, dorsal view; (2) Female lateral view; (3) Forewing; (4) Lower part of scutellum+dorsellum+ propodeum.

Head, mesosoma, and metasoma entirely black, all legs yellow except brown coxae (Fig. 1); malar sulcus not well developed (Fig. 2), clava 3—segmented, 3.8× as long as F4 with long terminal spine (Fig. 2); scutellum, dorsellem, and propodeum with similar reticulation; propodeum with median carina, callus with two setae (Fig. 4); forewing with SMV with three setae, PMV as stub, basal cell with four setae in distal margin (Fig. 3).

The following characters are added to the description of female *T. repanda* based on our material.

Female. Body length 0.72–0.75 mm.

Head 1.3× as broad as height. Eyes asetose. Malar sulcus not well developed. Mandibles with two big (chitin brown) teeth. POL 1.4× as OOL. Antenna (Fig. 2) with scape 3.5× as long as wide; pedicel 1.28× as long as wide, with one anellus; funicle with three transverse flagellomeres: F1 (1.5× as wide as long), F2 (2.2× as wide as long), F3 (2.2× as wide as long), clava 3—segmented, 1.8× as long as wide and 3.8× as long as F4. Terminal spine as long as one fourth of clava length.

Mesosoma. Pronotum 2.2× as broad as long. Mesoscutum 1.75× as broad as long, with three adnotular setae, median line absent. Scutellum 1.4× as broad as long, without submedian lines. Propodeum 3.6–3.7× as broad as long and 1.9× as long as dorsellum; with median carina, callus with two setae, sculpture seen in Fig. 4.

Forewing (Fig. 3) SMV with three to four setae; MV with eight to nine setae. Relative measurement: SMV: MV: STV = 40:62:19. PMV as stub. Speculum narrow and along ½ MV, closed. Basal cell with four setae in distal margin (Fig. 3). Hindwing apically rounded.

Metasoma. Petiole transverse. Gaster 1.1–1.2× as long as broad.

Color. Body black (Figs. 1 and 2). Head black; antenna brown, scape, pedicel dorsally pale brownish, eye red, ocelii white. All coxae and femorae brown, hind leg with brown tibia, other parts of legs yellow; gaster brown.

### Comments

Males were not found in this sample of 67 females. Bouček (1977) reported examining over 500 females and only 9 males, and Mayer and Shull (1978) found 69 females and 9 males. Thus, there is clearly a strongly female-biased sex ratio, as is often found in parasitoid wasps (Godfray 1994).

### Material Examined

Sixty-seven females, New Papua Guinea, Morobe Prov. 24 August of 1980, Wau Ecology Institute, Y. Lubin, parasitic wasps of *C. moluccensis* egg sacs (Museum of Comparative Zoology, Harvard University, Cambridge, USA; MCZ-ENT 00744591_Eulophidae).

### *Tachinobia repanda*—A Hyperparasitoid or Parasitoid of *S. (B.) cyrtophorae*?

The information on the museum label indicated the source of the 67 *T. repanda* specimens from PNG as egg sacs of the spider *C. moluccensis*; we are missing any additional biological notes, for example, on the number of wasps in each egg sac. *Tachinoba repanda* was never recorded as an egg parasitoid or egg predator, and previous records indicate that it is a gregarious hyperparasitoid of fly larvae that are parasitoids of a range of lepidopteran hosts (see Introduction). However, *T. repanda* was also recorded from a dipteran pupa (identity unknown) from a spider egg sac in Cuba (Bouček 1977) and from larvae of *S. banksi* Senior-White (Diptera: Sarcophagidae) in egg sacs of the orb-weaving spider *Argiope pulchella* Thorell (Araneae: Araneidae) in India (Prakash and Pandian 1978). Prakash and Pandian (1978) reported that egg sacs of *A. pulchella* were parasitized by the one to three larvae of the fly *S. banksi*, and 25–26% of these fly-parasitized egg sacs were parasitized by *T. repanda*. No *S. banksi* adults emerged when parasitized by *T. repanda*. These data suggest that the eulophid lays multiple eggs in larvae of *S. banksi* in early developmental stages, and the adult wasps emerge either from the last stage of the maggot or from the pupa.

Similar to the above study, we suggest that the hosts of the examined specimens of *T. repanda* from PNG were likely larvae of a sarcophagid fly parasitizing *C. moluccensis* egg sacs. Sarcophagid flies were observed searching webs that contained egg sacs in colonies of *C. moluccensis* (Lubin 1974). The egg sacs of *C. moluccensis* are hung in a string above the hub of a horizontal orb web, such that the most recent egg sac, usually containing fresh eggs, is the lowest one in the string and closest to the guarding female. Up to 22% of egg sacs contained sarcophagid puparia, and 6–7% were recorded as also containing parasitoid wasps of unknown identity (Lubin 1974).

In a later study (Y.L., unpublished data), contents of egg sacs from 18 *C. moluccensis* colonies in the vicinity of the Wau valley, PNG, were examined between January and October 1980. Puparia and adults of the sarcophagid fly, *S.* (*Baraniscova*) *cyrtophorae* (ID verified by Cantrell 1986; see also Cantrell 1981, Meiklejohn et al. 2013), were found in 19.8% of 313 egg sacs. Parasitoid wasps of unknown identity were found together with the fly larvae and puparia in four egg sacs collected on three dates (collection dates: 5.v.1980, 8.vii.1980, and 24.vii.1980). It is likely that the 67 specimens studied here are from these four egg sacs. Egg sacs of *C. moluccensis* are quite large (mean diameter ± SD, 18 ± 2.3 × 14 ± 1.4 mm, *n* = 15) and contain up to 1,000 eggs (876 ± 299, *n* = 4). The number of fly larvae or puparia in a single egg sac ranged from 1 to 10 (median = 3, *n* = 62; Y.L., unpublished data), and the third instar larvae are 8.5–11 mm long (Cantrell 1981), thus potentially providing an ample source of food for numerous wasp larvae. It is unknown how many wasps emerged from a single puparium. If we assume that the 67 specimens were from the four egg sacs noted above, then each egg sac yielded on average 16 wasps.

To successfully parasitize an egg sac of *C. moluccensis*, both of the parasitoids—the fly and the eulophid wasp—must overcome defenses of the spider. The female spider aggressively guards the egg sacs against the sarcophagid fly by shaking the web and circling her egg sacs (Lubin 1974). It is unknown if the spider detects the eulophid hyperparasitoid. Another sarcophagid fly, *Arachnidomyia lindae* Lopes, was observed to deposit (larviposit) a single first-instar larva on the egg sac of the colonial spider *Metepeira incrassata* F. O. Pickard-Cambridge (Araneidae; Hieber et al. 2002); presumably, the fly larva then burrows into the egg sac. The behavior of *S. (B.) cyrtophorae* may be similar, although with the fly often depositing more than a single larva on the egg sac. As *T. repanda* is a gregarious endoparasitoid, it is likely that it parasitizes this first-instar fly larva before the larva works its way into the spider’s egg sac. The sarcophagid fly larva consumes the spider eggs and pupates inside the egg sac. It is unknown how the emerging fly or its wasp parasitoids exit the dense silk of the egg sac.

The fly might be considered an egg predator rather than a parasitoid (where the latter is defined as an insect whose larvae develop on an arthropod body, resulting in death of the host [Godfray 1994]), in which case *T. repanda* is a parasitoid of the fly. However, the behavior of *S. (B.) cyrtophorae* vis à vis the *C. moluccensis* egg sac is identical to that of a parasitoid, whereby the egg sac is ‘hunted’ and attacked in a manner similar to an attack on a host larva. Accordingly, we regard *S. (B.) cyrtophorae* as a parasitoid and *T. repanda* as a hyperparasitoid of its dipteran host.
